# Body image and social appearance anxiety in patients with cancer undergoing radiotherapy: a cross-sectional study

**DOI:** 10.1186/s40359-024-01856-w

**Published:** 2024-06-24

**Authors:** Fatma Arıkan, Funda Kartöz, Zeynep Karakuş, Müge Altınışık, Zeynep Özer, Aylin Fidan Korcum Şahin

**Affiliations:** 1 Department of Internal Medicine Nursing, Faculty of Nursing, Akdeniz University, Dumlupınar Boulevard, Campus, Antalya, 07070 Türkiye; 2 Department of Radiation Oncology, Akdeniz University Hospital, Dumlupınar Boulevard, Antalya, 07059 Türkiye; 3 Department of Radiation Oncology, Faculty of Medicine, Akdeniz University, Dumlupınar Boulevard, Campus, Antalya, 07059 Türkiye

**Keywords:** Body image, Cancer patient, Social appearance anxiety

## Abstract

**Background:**

The body image of patients with cancer can be negatively affected due to treatment toxicities. Changes in body image may cause patients to experience social appearance anxiety. This study aimed to evaluate the body image and social appearance anxiety of patients with cancer undergoing radiotherapy.

**Methods:**

The cross-sectional study was conducted with 153 patients with cancer undergoing radiotherapy in a university hospital. The data were collected with a Patient Information Form, the Body Image Scale, and the Social Appearance Anxiety Scale and the Radiation Therapy Oncology Group Skin Toxicity Criteria.

**Results:**

Patients’ mean body image score was 15.18 ± 8.26 (min = 0, max = 30), mean social appearance anxiety score was 45.29 ± 14.50 (min = 16, max = 80). Patients with low education levels and low-income levels had higher body image and social appearance anxiety scores (*p* < 0.01). Body image and social appearance anxiety scores were found to be higher in patients with advanced cancer, grade III-IV skin toxicity, pain, fatigue, and constipation (*p* < 0.05).

**Conclusions:**

Radiotherapy may negatively affect body image and social appearance anxiety. Assessments of body image and social appearance anxiety regularly before, during, and after treatment are essential. Psychosocial support should be provided to patients to reduce body image and social appearance anxiety and increase their well-being. Patients with cancer especially those who have low income and education levels, advanced cancer stage and skin toxicity, and suffer from pain, fatigue, constipation, etc. should be supported by methods such as counseling and social support groups.

## Background

Cancer is one of the major burdens of disease and a leading cause of death worldwide [1]. Chemotherapy (CT), surgery, and radiotherapy (RT) are the mainstays of cancer treatment. These treatment modalities may cause severe side effects, including cardiotoxicity, nephrotoxicity, neurotoxicity, gastrointestinal toxicity, and skin toxicity [2]. Skin-related toxicities cause changes in the physical appearance of patients and directly affect their body image [3, 4]. A previous study determined that skin toxicity experienced by patients with cancer increased anxiety of patients related to their appearance and body image [5].

Body image is a multidimensional concept that involves individuals’ social and psychological experiences influenced by their perceptions of their appearance [6, 7]. It is frequently described as the fixed mental image of one’s physical appearance influenced by one’s view of oneself and the reactions of others [8]. Physical appearance is associated with body image [9]. Due to anticancer treatments like RT, changes that have temporary or permanent effects occur in the appearance of patients with cancer [10]. RT may cause burns, skin irritation, edema, scars, and inflamed wounds which may cause psychological distress [11]. Melissant et al. [12] found that changes in appearance and dysfunction were related to negative body image experiences. Changes in appearance and body image can cause social appearance anxiety in patients with cancer [13]. Social appearance anxiety is defined as anxiety about being negatively evaluated by people regarding their overall appearance, including body shape [14]. The significant impact of appearance-related side effects on the body image of patients requires a better understanding of social appearance anxiety. Healthcare providers should consider the presence of risk factors for poor body image and social appearance anxiety [15].

In the literature, some studies examining especially body image in patients with cancer are available [16, 17]. Studies mainly focused on the body image of younger patients who have specific cancer such as breast or head and neck cancers or social appearance anxiety of adolescents. However, to our knowledge, body image and social appearance anxiety have not been studied together in patients with cancer undergoing RT. More data are needed to understand the body image and social appearance anxiety of patients with cancer undergoing RT. This study aimed to evaluate the body image and social appearance anxiety of patients with cancer undergoing RT.

## Methods

### Design

This cross-sectional study was conducted between December 2018 and January 2019 in a radiation therapy unit of a university hospital.

### Participants and data collection

The study inclusion criteria were the following: (a) patients with cancer undergoing RT, (b) those over the age of 18, and (c) those with a person-place-time orientation. Patients who had speech difficulties due to RT complications (dry mouth, mucositis, decreased salivation, pain, nausea-vomiting, etc.) and who did not want to participate in the study were not included. The data were collected by the researchers during the nurse visit to the RT unit. The researcher nurses collected the data by using Patient Information Form, Body Image Scale (BIS), Social Appearance Anxiety Scale (SAAS) and the Radiation Therapy Oncology Group (RTOG) Skin Toxicity Criteria. The skin toxicity was assessed by the second author (F.K.) who works as a RT nurse. The participants were explained about the study and an informed consent form was obtained. The data were collected by pen-and-paper questionnaire.

## Measures

### Patient information form

The Patient Information Form was prepared by the researchers with reference to the literature [18–20]. It includes information about patients’ sociodemographic characteristics (age, gender, marital and educational status, etc.) and disease characteristics (cancer type, symptoms, etc.).

### Body image scale

The Body Image Scale was developed by Hopwood et al. [21] for a brief and complete evaluation of cancer patients’ body image. It is a 4-point Likert-type scale consisting of 10 items. The lowest score that can be obtained from the scale is 0, while the highest is 30. A low score indicates a better body image. In the Turkish validity and reliability study, Cronbach’s alpha coefficient was found to be 0.94 [22]. In this study, Cronbach’s alpha coefficient of the scale was 0.92.

### Social appearance anxiety scale

The Social Appearance Anxiety Scale was developed by Hart et al. [14] with the aim of assessing the social appearance anxiety. The self-report scale consists of 16 items ranging from 1 (not at all) to 5 (extremely) [23]. The lowest score that can be obtained from the scale is 16, while the highest is 80 and high scores indicate high appearance anxiety. Scores of 16–37 are assessed as “low” anxiety, 38–59 as “moderate” anxiety, and 60–80 as “high” anxiety [14]. In the Turkish validity and reliability study of the scale, the internal consistency coefficient was found to be 0.93 [23]. In this study, Cronbach alpha coefficient was to be 0.94.

### Radiation therapy oncology group skin toxicity criteria

Skin toxicity was assessed by using the Skin Toxicity Criteria of the RTOG with scores ranging from 0 to 4 (0 = none; 1 = slight atrophy, change in pigmentation, some hair loss; 2 = patch atrophy, moderate telangiectasia, total hair loss; 3 = market atrophy, gross telangiectasia; 4 = ulceration) [24].

## Ethical considerations

Permission was obtained from the clinical research ethics committee of the university (No:70904504/479) and the hospital where the study was conducted. The study was conducted in accordance with the Declaration of Helsinki. Written and oral consent was obtained from participants of the study.

### Data analysis

The data analysis was performed using the Statistical Package for the Social Sciences (SPSS) Statistics Base v23 software program (IBM SPSS Corp. Armonk, NY, USA). Variables were tested for normality using Shapiro-Wilk and skewness test. The descriptive statistics were presented with frequencies, percentages, means and standard deviations. T-test analysis was used for examining the dimensions according to two groups and analysis of variance was performed for comparison of more than two groups. The Sidak pairwise comparison test was applied to determine groups that were different according to variance analysis. Pearson correlation analysis was conducted to determine the relationship between variables. Cronbach’s alpha analysis was used for testing the internal consistency of the dimensions. The statistical significance level was considered *p* < 0.05 in the study.

## Results

### Patients’ characteristics

The study sample comprised a total of 153 patients. The median age of the patients was 59.2. Of the patients 52.9% were male, 85.6% were married, and 29.4% had breast cancer, 28.1% had other cancer such as gastrointestinal, urogenital cancer, 20.3% had lung cancer. Previous treatments of the patients were 73.2% CT, 17.6% RT and 66% surgery. The most common symptoms of patients were fatigue and pain (75.8% and 65.4%, respectively). In 42% of the patients grade II skin toxicity was found (Table 1).

**Table 1:** Sociodemographic and disease characteristics of participants (*n* = 153)

Characteristics			*n*	%
**Age**	54 and under		60	39.2
	55–65 age		53	34.6
	66 and over		40	26.2
	*Median (years) = 59.2*			
**Gender**	Female		72	47.1
	Male		81	52.9
**Marital status**	Married		131	85.6
	Single		22	14.4
**Education status**	Illiterate		13	8.5
	Primary -secondary education		119	77.8
	High education		21	13.7
**Income and expenditure status**	Income is less than expenses		88	57.5
	Income coincides with expenses		57	37.3
	Income is more than expenses		8	5.2
**Cancer type**	Breast		45	29.4
	Lung		31	20.3
	Head and neck		34	22.2
	Other *		43	28.1
**Cancer stage**	Stage I		21	13.7
	Stage II		42	27.5
	Stage III		29	19.0
	Stage IV		61	39.9
**Radiotherapy**	Adjuvant		94	61.4
	Curative		25	16.3
	Palliative		34	2.2
Previous treatments**	Chemotherapy	Yes	112	73.2
		No	41	26.8
	Radiotherapy	Yes	27	17.6
		No	126	82.4
	Surgery	Yes	101	66.0
		No	52	34.0
Symptoms**	Pain	Yes	100	65.4
		No	53	36.6
	Fatigue	Yes	116	75.8
		No	37	24.2
	Diarrhea	Yes	22	14.4
		No	131	85.6
	Constipation	Yes	41	26.8
		No	112	73.2
	Nausea	Yes	38	24.8
		No	115	75.2
	Alopecia	Yes	79	51.6
		No	74	48.4
**Skin Toxicity**	No symptoms		18	11.8
	Grade I		47	30.7
	Grade II		65	42.5
	Grade III		17	11.1
	Grade IV		6	3.9

^*^Gastrointestinal, urogenital, hematological, and gynecological cancer, **more than one choice was selected

### Body image

Patients’ mean body image score was 15.18 ± 8.26 (min = 0, max = 30) (Table 2). Patients with low education levels (illiterate) and low-income levels had higher body image score (*p* < 0.01). Body image score was found to be higher in patients with advanced cancer, grade III skin toxicity, pain, fatigue, and constipation, and those who had received RT before and who received palliative RT (*p* < 0.05). A higher body image score indicates that these patients have worse body image (Table 3).

**Table 2:** Body image and social appearance anxiety scores

	Mean	SD	Min-Max
Body image	15.18	8.26	0–30
Social appearance anxiety	45.29	14.50	16–80

Abbreviation: SD, Standard deviation

**Table 3:** Comparison of body image and social appearance anxiety by sociodemographic and disease characteristics

			Body Image			Social Appearance Anxiety		
			Mean	SD	*p*	Mean	SD	*p*
**Gender**	Female		15.01	8.81	0.81	44.28	15.01	0.42
	Male		15.33	7.78		46.20	14.07	
**Age**	54 and under		14.35	8.70	0.59	43.73	16.12	0.47
	55–65		15.48	8.36		47.24	13.62	
	66 and over		15.96	7.63		45.38	13.16	
**Education status**	Illiterate^a^		18.85	9.66	< **0.01**	47.77	13.85	< **0.01**
	Primary-secondary education^b^		15.71	7.51		46.80	13.83	
	High education^c^		9.95	9.53		35.24	15.20	
			**a > b,c**			**a > b,c**		
**Marital status**	Married		15.18	8.08	0.98	45.02	14.39	0.57
	Single		15.23	9.47		46.91	15.38	
**Income and expenditure status**	Income less than expenses^a^		17.24	7.73	< **0.01**	48.25	13.86	< **0.01**
	Income coincides with expenses^b^		12.67	8.05		42.44	14.62	
	Income more than expenses^c^		10.50	9.46		33.13	11.22	
			**a > b,c**		**a > b,c**			
**Cancer type**	Breast		14.20	8.51	0.73	42.06	14.97	0.30
	Lung		14.88	9.58		45.23	16.81	
	Head and neck		18.09	6.18		47.74	11.61	
	Other^*^		15.79	8.37		46.95	13.76	
**Cancer stage**	Stage 1^a^		10.24	8.29	< **0.01**	38.19	14.02	< **0.01**
	Stage 2^b^		12.10	8.44		39.07	14.41	
	Stage 3^c^		15.17	8.40		44.76	15.05	
	Stage 4^d^		19.02	6.18		52.28	11.19	
			**d > a,b, c**			**d > a,b, c**		
**Radiotherapy**	Adjuvant^a^		14.28	8.48	<**0.001**	43.59	14.79	< **0.001**
	Curative^b^		13.24	8.06		40.88	12.75	
	Palliative^c^		19.08	6.56		53.23	12.08	
			**c > a,b**			**c > a,b**		
**Previous treatments**	Chemotherapy	Yes	15.26	8.38	0.85	45.80	14.64	0.47
		No	14.98	8.01		43.90	14.21	
	Radiotherapy	Yes	18.52	7.29	< **0.02**	54.26	12.36	< **0.01**
		No	14.47	8.30		43.37	14.24	
	Surgery	Yes	15.75	8.12	0.24	46.31	14.56	0.23
		No	14.08	8.48		43.33	14.33	
**Symptoms**	Pain	Yes	17.10	8.05	< **0.01**	47.35	14.71	< **0.02**
		No	11.57	7.46		41.42	13.39	
	Fatigue	Yes	16.91	7.97	< **0.01**	48.18	13.49	< **0.01**
		No	9.76	6.71		36.24	13.98	
	Diarrhea	Yes	17.27	7.60	0.20	45.14	15.46	0.96
		No	14.83	8.34		45.32	14.40	
	Constipation	Yes	18.10	7.27	< **0.01**	50.46	12.64	< **0.01**
		No	14.12	8.37		43.40	14.73	
	Nausea	Yes	17.58	6.94	0.05	49.03	14.53	0.07
		No	14.39	8.53		44.06	14.34	
	Alopecia	Yes	15.95	7.78	0.24	45.57	14.64	0.81
		No	14.36	8.72		45.00	14.45	
**Skin Toxicity**	No symptoms^a^		9.27	6.70	<**0.001**	35.77	13.12	< **0.001**
	Grade I^b^		13.12	7.45		42.48	14.08	
	Grade II^c^		16.83	7.70		47.79	13.11	
	Grade III^d^		19.52	7.72		51.52	16.32	
	Grade IV^e^		18.83	8.13		54.33	15.37	
			**d > a,b, c,e**			**e > a,b, c,d**		

^*^Gastrointestinal, urogenital, hematological, and gynecological cancer; Abbreviations: SD, Standard deviation

### Social appearance anxiety

Patients’ mean social appearance anxiety score was 45.29 ± 14.50 (min = 16, max = 80) (Table 2). Patients with low education levels (illiterate) and a low-income level had higher social appearance anxiety score (*p* < 0.01). Social appearance anxiety score was found to be higher in patients with advanced cancer, grade IV skin toxicity, pain, fatigue, and constipation, and those who had received RT before and who received palliative RT (*p* < 0.05). The increase in the score indicates that the social appearance anxiety of these patients increases (Table 3).

### Relationship between body image, social appearance anxiety, and skin toxicity

A moderately significant positive relationship was found between skin toxicity and body image and social appearance anxiety. A strong positive relationship was determined between body image and social appearance anxiety (*p* < 0.05) (Table 4).

**Table 4:** Correlations of body image, social appearance anxiety, and skin toxicity

		Skin toxicity	Body image
**Body image**	r	0.35*	
	p	0.01	
**Social appearance anxiety**	r	0.33*	0.78*
	p	0.01	0.01

*Pearson correlation analysis

## Discussion

This study examined the body image and social appearance anxiety of patients with cancer undergoing RT. The results of this study showed that education and income level, cancer stage and skin toxicity, pain, fatigue and constipation, receiving prior RT and palliative therapy affected body image and social appearance anxiety. Moreover, a strong positive relationship was determined between body image and social appearance anxiety in the study.

Socioeconomic status is important for a person’s attitudes towards his/her own appearance, and a poor socioeconomic status can be devastating for patients with cancer [25, 26]. Patients with poor levels of education and income may have difficulty accessing information, which may make it difficult for them to manage the side effects of cancer and the treatments. They may also have trouble adapting to changes in their appearance and functionality [8, 27]. In our study, it was found that patients with low-education and income level had worse body image and social appearance anxiety. Similar to our study, some previous studies have associated poor body image with low education and income status [28–30]. However, some previous studies did not find a significant relationship between income and education status for body image [27, 31]. Also, Chen et al. [27] found that patients with higher education had greater body image dissatisfaction. It is important data that patients with low education and income level are riskier in terms of body image and physical image anxiety. It is thought that these patients need more support to cope with the deterioration in their appearance caused by cancer treatment due to the lack of resources such as not being able to buy cosmetic products due to poverty, and lack of knowledge about cosmetic improvement.

Changes in physical appearance are a symbol of the disease for many patients and affect their relationships in social life by causing patients to experience social appearance anxiety [32]. The advanced cancer stage generally requires more complicated treatments, resulting in greater changes in appearance [33]. In our study, it was found that patients with advanced cancer stage, pain, fatigue and constipation, those who had received RT before and those who received palliative RT had worse body image and social appearance anxiety. Similar to our study, some previous studies concluded that the advanced cancer stage was associated with poorer body image [27, 34]. In a study, it was found that body image concerns were associated with physical symptom burden upon being diagnosed with cancer and immediately post-treatment [35]. It is thought that the more complex treatment and more intense symptoms of advanced patients with cancer may increase body image concerns even after treatment. On the other hand, the changes in appearance due to cancer or treatment may be raising also existential concerns about advanced cancer [36]. It is recommended that qualitative studies be carried out in order to understand the two-way connections between cancer stage and body image and social appearance anxiety in depth.

Skin changes due to RT negatively affect the body image and appearance of the patients. Changes in patients’ appearances lead to anxiety about their social appearance by affecting their physical and social functions [37, 38]. In a qualitative study, patients reported that they were worried that the changes occurring in their skin might become the subject of gossip by other people [5]. In our study, in accordance with the literature, it was found that patients with advanced skin toxicity had worse body image and social appearance anxiety. When they provide care to a patient experiencing changes occurring in his/her body, nurses should be supportive with regard to the potential effects of these changes on the body, mind, and soul as well as social relationships. Therefore, it is important to regularly monitor body image and social appearance anxiety that may occur in patients, and to begin early rehabilitation programmes.

### Study limitations

The fact that the sample was from a single hospital may have affected the generalizability of the research results. One of the limitations of this study is that the study results only reflect data during RT. In the future, it is recommended that researchers should consider body image and appearance concerns comparatively in patients before, during, and after RT. Another limitation is that there were no additional measures, such as assessing whether patients utilized strategies to enhance their body image such as makeup, or had adequate social support to address image concerns had not been evaluated in this study. Therefore, we recommend that these aspects could be explored in future studies.

## Conclusions

Radiotherapy often causes skin-related problems such as burns, skin irritations, and scars, therefore, patients may have body image disturbances and social appearance anxiety following these changes. As a result of this study, education and income level, cancer stage and skin toxicity, pain, fatigue and constipation, previous RT and palliative treatment status affected body image and social appearance anxiety. Patients with cancer (low income and education level, advanced cancer stage and skin toxicity, and suffering from pain, fatigue, constipation etc.) should be supported early to manage and minimize the devastating effects of cancer treatment especially RT on their bodies. Counseling and social support groups can reduce the patient’s social appearance anxiety.

This study contributes valuable information on body image and social appearance anxiety in patients with cancer undergoing RT and provides data for future studies aimed at improving body image and social appearance anxiety in these patients. All patients with cancer should be supported especially who are at risk in terms of body image and social appearance anxiety, in the early period of RT. It is also suggested that body image and social appearance anxiety should be regularly assessed at the beginning and following RT. It is also recommended that patients be supported regarding body image and social appearance anxiety after RT.

## Data Availability

The datasets are available from the corresponding author upon reasonable request for research purposes.

## Abbreviations

CT: Chemotherapy
RT: Radiotherapy
RTOG: Radiation Therapy Oncology Group
SPSS: Statistical Package for the Social Sciences
BIS: Body Image Scale
SAAS: Social Appearance Anxiety Scale
SD: Standard Deviation
