# Correction: Body image and social appearance anxiety in patients with cancer undergoing radiotherapy: a cross-sectional study

**DOI:** 10.1186/s40359-024-01893-5

**Published:** 2024-07-15

**Authors:** Fatma Arıkan, Funda Kartöz, Zeynep Karakuş, Müge Altınışık, Zeynep Özer, Aylin Fidan Korcum Şahin

**Affiliations:** 1 Department of Internal Medicine Nursing, Faculty of Nursing, Akdeniz University, Dumlupınar Boulevard, Campus, Antalya, 07070 Türkiye; 2 Department of Radiation Oncology, Akdeniz University Hospital, Dumlupınar Boulevard, Antalya, 07059 Türkiye; 3 Department of Radiation Oncology, Faculty of Medicine, Akdeniz University, Dumlupınar Boulevard, Campus, Antalya, 07059 Türkiye

**BMC Psychology (2024) 12:363**

10.1186/s40359-024-01856-w

Following publication of the article, it came to the authors’ attention that the wrong number had been provided in the ‘Ethics approval and consent to participate’ declaration. In place of ‘No:70904504/479’, the correct number, ‘No:70904504/721’ had been provided. The number has since been corrected in the published article. The authors thank you for reading this erratum and apologize for any inconvenience caused.

